# Effects of Fluoxetine on Human Embryo Development

**DOI:** 10.3389/fncel.2016.00160

**Published:** 2016-06-16

**Authors:** Helena Kaihola, Fatma G. Yaldir, Julius Hreinsson, Katarina Hörnaeus, Jonas Bergquist, Jocelien D. A. Olivier, Helena Åkerud, Inger Sundström-Poromaa

**Affiliations:** ^1^Department of Women's and Children's Health, Uppsala UniversityUppsala, Sweden; ^2^Centre of Reproduction, Uppsala University HospitalUppsala, Sweden; ^3^IVF-Clinic FalunFalun, Sweden; ^4^Analytical Chemistry, Department of Chemistry – BMC and Science for Life Laboratory, Uppsala UniversityUppsala, Sweden; ^5^Unit Behavioural Neuroscience, Department of Neurobiology, Groningen Institute for Evolutionary Life Sciences, University of GroningenGroningen, Netherlands

**Keywords:** embryo development, selective serotonin reuptake inhibitors, serotonin, human, time-lapse monitoring, proteomics, secretomics, shotgun mass spectrometry

## Abstract

The use of antidepressant treatment during pregnancy is increasing, and selective serotonin reuptake inhibitors (SSRIs) are the most widely prescribed antidepressants in pregnant women. Serotonin plays a role in embryogenesis, and serotonin transporters are expressed in two-cell mouse embryos. Thus, the aim of the present study was to evaluate whether fluoxetine, one of the most prescribed SSRI antidepressant world-wide, exposure influences the timing of different embryo developmental stages, and furthermore, to analyze what protein, and protein networks, are affected by fluoxetine in the early embryo development. Human embryos (*n* = 48) were randomly assigned to treatment with 0.25 or 0.5 μM fluoxetine in culture medium. Embryo development was evaluated by time-lapse monitoring. The fluoxetine-induced human embryo proteome was analyzed by shotgun mass spectrometry. Protein secretion from fluoxetine-exposed human embryos was analyzed by use of high-multiplex immunoassay. The lower dose of fluoxetine had no influence on embryo development. A trend toward reduced time between thawing and start of cavitation was noted in embryos treated with 0.5 μM fluoxetine (*p* = 0.065). Protein analysis by shotgun mass spectrometry detected 45 proteins that were uniquely expressed in fluoxetine-treated embryos. These proteins are involved in cell growth, survival, proliferation, and inflammatory response. Culturing with 0.5 μM, but not 0.25 μM fluoxetine, caused a significant increase in urokinase-type plasminogen activator (uPA) in the culture medium. In conclusion, fluoxetine has marginal effects on the timing of developmental stages in embryos, but induces expression and secretion of several proteins in a manner that depends on dose. For these reasons, and in line with current guidelines, the lowest possible dose of SSRI should be used in pregnant women who need to continue treatment.

## Introduction

The prevalence of major depression during pregnancy is approximately 3–5% (Andersson et al., 2003; Gavin et al., 2005). Nowadays, selective serotonin reuptake inhibitors (SSRIs) are the most widely prescribed antidepressants in pregnant women as these drugs, besides proven efficacy, are associated with fairly few side-effects. While generally also considered safe to use during pregnancy (Barbey and Roose, 1998; Gentile, 2005), SSRI treatment has been associated with increased risk of pregnancy complications such as preterm birth and preeclampsia (Qiu et al., 2009; Wisner et al., 2009). With regards to the offspring, reduced fetal head growth and low birth weight have been reported (Kallen, 2004; Wen et al., 2006; El Marroun et al., 2012). SSRIs have also been associated with cardiac malformations (Olivier et al., 2013; El Marroun et al., 2014; however, see also Furu et al., 2015) and increased risk of persistent pulmonary hypertension, although these conditions are rare (Wogelius et al., 2006; Kieler et al., 2012). Behavioral defects caused by antenatal SSRI exposure include increased risk for autism spectrum disorders and externalizing behaviors (Oberlander et al., 2007; Croen et al., 2011). However, other studies suggest that these malformations and behavioral defects may be caused by the underlying depression and not by the treatment itself (reviewed in Olivier et al., 2013, 2015a; Waters et al., 2014).

SSRIs inhibit the reuptake of released serotonin (5-HT) by blocking the serotonin transporter, which results in increased levels of 5-HT in the synaptic cleft. Serotonin is important during brain development, where it acts as a neurotrophic factor (Lauder et al., 1981; Gaspar et al., 2003; Ansorge et al., 2007). Lauder and Krebs (1978) found that serotonin is involved in early neurogenesis in chicken embryo and it has been shown in mice that 5-HT produced by the placenta is accumulated in the fetal forebrain during critical periods of brain development (Bonnin and Levitt, 2012). 5-HT is also important for the regulation of embryogenesis in such diverse species like sea urchins and mice (reviewed in Buznikov et al., 2001), where high and low levels of serotonin can have both positive and negative effects, depending on the stage of development (Khozhai et al., 1995; Il'kova et al., 2004). Of relevance for the research question at hand, serotonin transporters are expressed as early as in two-cell mouse embryos (Amireault and Dube, 2005). In addition, serotonin plays a crucial role during craniofacial development (Moiseiwitsch and Lauder, 1995) and cardiac morphogenesis in mice (Yavarone et al., 1993). SSRIs are found in cord blood and amniotic fluid of SSRI-treated pregnant women (Hendrick et al., 2003; Loughhead et al., 2006), indicating that the fetus is exposed to SSRIs during pregnancy.

In previous studies, we have shown that SSRI treatment affects gene expression and protein levels in human placenta (Kaihola et al., 2015; Olivier et al., 2015b). While these effects were minor (at least in terms of fold change) in comparison to what is typically seen in preeclampsia or other placental disorders (Winn et al., 2009), it may be speculated that the changes we see, for instance in nerve growth factor signaling (Kaihola et al., 2015), may influence placental function, and thus, the intrauterine development of the fetus. To further investigate the impact of SSRIs on fetal development, studies focusing on embryonic development before implantation are needed. The aim of the present study was to evaluate whether fluoxetine, one of the most widely prescribed SSRI antidepressants, exposure influences the timing of different embryo developmental stages, and furthermore, to analyze what protein, and protein networks, are affected by fluoxetine in the early embryo development.

## Materials and methods

All the couples who were asked to participate attended the Center for Reproduction, Uppsala University Hospital, Uppsala, Sweden, and written informed consent was obtained from all couples donating embryos for this study. No reimbursement was given to couples participating.

### Embryo collection, culturing, and developmental scoring

Couples who had undergone *in vitro* fertilization (IVF) treatment at the Center for Reproduction, Uppsala, Sweden, were asked to donate surplus cryopreserved embryos that otherwise, according to Swedish law, had to be destroyed following 5 years of cryopreservation.

Before freezing the oocytes were inseminated with sperm in IVF medium (G-IVF Plus™) (catalog no. 10136, Vitrolife, Sweden) and transferred to G1 medium (G1 Plus™) (catalog no. 10128, Vitrolife, Sweden) after evaluation of fertilization. Only zygotes with two pro-nuclei were selected for further culture. Embryos were cultured in 25 μL droplets of medium under an oil overlay, in a humidified incubator at 37°C and 6% CO_2_/6% O_2_.

All in all, 48 human embryos were used and no embryos were obtained from the same couple. The experiments were performed in two parts: (1) six embryos in each group were used for mass spectrometry analysis (see below) and (2) 10 embryos in each group were used for protein detection in culture medium and for immunofluorescent staining (see below). Cryopreserved 2-day embryos were thawed using a thawing kit (Sydney IVF Thawing Kit) (catalog no. G19014 Cook Medical Inc., US) and transferred to equilibrated culture medium used for human cleavage stage embryos (CCM) (catalog no. 10093, Vitrolife, Sweden) (Hambiliki et al., 2013). An independent embryologist scored the embryos and ensured that the three treatment groups would be as similar as possible at baseline. Following this group allocation, each group was randomly assigned to treatment with 0.25 or 0.5 μM fluoxetine or control. The fluoxetine doses were chosen based on the literature, information on maternal SSRI concentrations from one of our studies, and a pilot study. The literature suggested that 0.25 and 0.5 μM fluoxetine are within the ranges found in amniotic fluid of women treated with fluoxetine during pregnancy (Hendrick et al., 2003; Rampono et al., 2004; Loughhead et al., 2006). According to yet unpublished findings, fluoxetine concentrations in late pregnant women vary between 0.1 and 0.7 μmol/L, which should approximate the concentration to which the embryo is exposed. Finally, in a pilot study we found that embryos treated with 1.0 μM fluoxetine (*n* = 3) tended to develop faster but also die to a higher degree than control embryos.

For treatment with fluoxetine, fluoxetine was added to the culture medium to reach the concentrations of 0.25 or 0.5 μM fluoxetine (catalog no. F132, Sigma-Aldrich Corp., US), while control embryos were cultured in CCM medium without additives. All embryos were treated using the same batch of culturing medium, and each experiment included a blank media. The embryos were then cultured using time-lapse monitoring in an EmbryoScope® (Unisense FertiliTech, Denmark) for 4 days (i.e., until day six after insemination) in 6% CO_2_ and 6% O_2_ at 37°C (Hambiliki et al., 2013). Images of the embryos were recorded at 15 min intervals. The embryo quality was evaluated retrospectively using standard morphological criteria for cleavage stage embryos, according to Alpha Scientists in Reproductive Medicine and ESHRE Special Interest Group of Embryology (2011). The scoring was performed by an independent observer. Timing of different embryo developmental stages was determined as time after thawing, e.g., time to first division after thawing of the embryo. Embryos from both experiments were used for evaluation (*n* = 16 in each group). The cut off value for a high-quality embryo was the same as for blastocysts selected for transfer to the women, according to Alpha Scientists in Reproductive Medicine and ESHRE Special Interest Group of Embryology (2011).

### Shotgun mass spectrometry of embryos

After the embryos had been cultured, the medium was collected and saved for further analyses. The embryos (six embryos per condition) were snap frozen and run individually in a shotgun mass spectrometry analysis, for the detection of proteins expressed in the embryos. One embryo per condition was used for a pilot study and the data presented in the results are based on the five remaining embryos.

Embryonic samples were dissolved in four times the sample volume of 2 M thiourea (catalog no. 107979, Merck KGaA, Germany) /6 M urea (catalog no. 108488, Merck KGaA, Germany) in 50 mM ammonium bicarbonate (catalog no. 09830, Fluka, Germany). Cell lysis and protein extraction was performed by vigorous vortexing followed by 2 h incubation at −20°C and a final step of vigorous vortexing. The proteins were reduced with dithiothreitol (DTT) (catalog no. D5545, Sigma-Aldrich Corp., US) and alkylated with iodoacetamide (IAA) (catalog no. I1149, Sigma-Aldrich Corp., US) prior to enzymatic digestion. The digestion was performed using two different enzymes. First, the proteins were digested with Lys-C (catalog no. 129-02541, Wako Chemicals GmbH, Germany) for 2 h at room temperature (RT). Thereafter the urea concentration was diluted to < 2 M using 50 mM ammonium bicarbonate, and trypsin (catalog no. V5111, Promega, US) was added to the samples. The tryptic digestion was carried out at 37°C overnight. Prior to the analysis by mass spectrometry the peptides were purified by ZipTips® (catalog no. ZTC18SO96, Merck Millipore, US) (Bergquist et al., 2002) and dried in a SpeedVac® system.

All analyses were performed using a QExactive Plus Orbitrap mass spectrometer (Thermo Fisher Scientific, Germany) equipped with a nano electrospray ion source. Samples were dissolved in water/ formic acid (0.1%) (catalog no. 100264, Merck KGaA, Germany), and peptides were separated by reversed phase liquid chromatography using an EASY-nLC 1000 system (Thermo Fisher Scientific, Germany). A set-up of pre-column and analytical column was used. The pre-column was a 2 cm EASY-column (1D 100 μm, 5 μm C18) (catalog no. SC001, Thermo Fisher Scientific, Germany) and the analytical column was a 10 cm EASY-column (ID 75 μm, 3 μm, C18) (catalog no. SC2003, Thermo Fisher Scientific, Germany). Peptides were eluted with a 150 min linear gradient from 4 to 100% acetonitrile at 250 nL/min. The mass spectrometer was operated in positive ion mode, acquiring a survey mass spectrum with resolving power 70,000 and consecutive high collision dissociation (HCD) fragmentation spectra of the 10 most abundant ions.

The acquired data (RAW-files) were submitted to the Mascot™ search algorithm (Matrix Science, UK) embedded in Proteome Discoverer software (Version 1.4.0.288, Thermo Fisher Scientific, Germany) and searched against human proteins in the UniProtKB/Swiss-Prot database. The search parameters included: maximum 10 ppm and 0.02 Da error tolerance for the survey scan and MS/MS analysis, respectively; enzyme specificity was trypsin; maximum two missed cleavage sites were allowed; cysteine carbamidomethylation was set as static modification; oxidation (M) and deamidation (N,Q) were set as variable modifications. The protein identifications were based on at least two matching peptides of 95% confidence per protein.

### Immunofluorescent staining of embryos

After culturing, two high-quality embryos from each group were briefly washed in sterile phosphate-buffered saline (PBS) containing 0.8 mg/mL polyvinylpyrrolidon (PVP) Clinical Grade (catalog no. 1090500, MediCult, Origio, Denmark), fixated in 2.5% paraformaldehyde in PBS for 15 min at RT and then stored in PBS/PVP at 4°C. The embryos were permeabilized in 0.25% Triton X-100 in PBS/PVP for 30 min at RT, blocked in blocking solution containing 0.1% bovine serum albumin (BSA) and 0.01% Tween20 in PBS for 15 min at RT, and incubated with primary antibody (diluted 1:100 in blocking solution) overnight at 4°C. Primary antibodies against urokinase-type plasminogen activator (uPA) (catalog no. sc-6830) and nerve growth factor (NGF) (catalog no. sc-548) were used, both from SantaCruz Biotechnology Inc., US. After incubation, the embryos were washed 3 × 15 min in blocking solution, incubated with secondary antibodies (diluted 1:100 in blocking solution) for 1 h at RT and washed again 3 × 15 min in blocking solution. The secondary antibodies were labeled with fluorophores AlexaFluor488 and AlexaFluor594 (catalog no. A-21206 and A-11058, Life Technologies, Thermo Scientific Inc., US). After washing, the embryos were mounted on a slide in 5 μL of VectaShield (Vector Laboratories Inc., US) surrounded by Vaseline. A cover slip was lowered onto the slide, gently pressed against the Vaseline, sealed with nail varnish and allowed to dry. Pictures were taken using a fluorescence microscope (20x objective; Axio Observer.Z1, Carl Zeiss AG Corp. Germany).

### Protein detection in embryo culture medium

After embryo culturing, 20 μL of the culture medium was snap frozen (*n* = 10 in each group) and protein analysis was run on each individual sample by using Proseek Multiplex Immunoassay analysis based on Proximity Extension Assay Technology (Olink Bioscience, Sweden) (Lundberg et al., 2011; Assarsson et al., 2014). The Proseek Multiplex Inflammation I^96×96^ panel was chosen for this study and the analysis was run at Olink Bioscience facilities in Uppsala, Sweden.

### Statistical analyses

For analysis of proteins detected in the embryo, a Venn diagram was used to elucidate which proteins were uniquely expressed in each fluoxetine dose group and which proteins were detected in both fluoxetine groups (http://bioinfogp.cnb.csic.es/tools/venny/index.html). QIAGEN's Ingenuity Pathway Analysis (IPA®) (QIAGEN, Redwood City, US; www.qiagen.com/ingenuity) was used to determine which pathways the proteins of interest were involved in. IPA computes a score for each network according to the fit of that network to the user-defined set of focus proteins. The score, derived from a *p*-value, indicates the likelihood of the association between the focus proteins and a given pathway.

The timing of developmental stages, the ability to form a blastocyst, the quality of the embryos, as well as protein levels were compared by use of Mann-Whitney *U*-test. All statistical analyses were performed by the IBM Statistical Package for the Social Sciences 20.0 (IBM Corp., Armonk, US) for Windows software package.

### Ethical approval

The study was approved by the Regional Ethical Review Board in Uppsala, Sweden (2014/298).

## Results

### Embryo development

The impact of fluoxetine on embryo development was evaluated. Human embryos exposed to 0.25 or 0.5 μM fluoxetine were compared to embryos cultured in control medium (*n* = 16 in each group). The embryos were cultured for 4 days under conditions similar to those used in assisted reproduction and were monitored by a time-lapse system. No differences between treatment groups were noted at baseline (Table 1). Embryos treated with 0.25 μM fluoxetine reached the individual developmental stages after thawing at almost the same time as control embryos. The embryos treated with 0.5 μM fluoxetine tended to develop more quickly, with a shorter time for development to start of cavitation after thawing (*p* = 0.065; Table 1). No significant differences in the ability to form a blastocyst or the proportion of high-quality embryos were noted between fluoxetine supplement and the control conditions (Table 1).

**Table 1:** **Embryo demographics at thawing, timing of different developmental stages after thawing, the number of embryos that developed into blastocysts, and the number of high-quality embryos following treatment with 0.25 or 0.5 μM fluoxetine**.

**Embryo demographics and time difference from thawing (hours)**	**Control *n*^a^ = 16 median (IQR)**	**0.25 μM fluoxetine *n*^a^ = 16 median (IQR)**	**0.5 μM fluoxetine *n*^a^ = 16 median (IQR)**
Number of cells at thawing, n	4 (4–4)	4 (4–4.8)	4 (4–5.5)
Number of embryos with ≥ 75% live cells at thawing, n (%)	15 (93.8%)	16 (100%)	15 (93.8%)
Number of embryos with 100% live cells at thawing, n (%)	10 (62.5)	10 (62.5)	11 (68.8%)
First cell division, h	9.8 (4.7–16.7)	8.1 (2.5–21.2)	11.8 (4.8–14.5)
Fourth cell division, h	33.4 (13.8–41.4)	30.6 (21.2–38.8)	22.5 (17.0–30.1)
Compaction, h	52.8 (45.0–60.3)	53.0 (41.0–63.4)	47.8 (40.6–55.3)
Cavitation, h	65.1 (53.3–70.8)	60.4 (50.1–72.6)	54.0 (43.6–63.5)^a^
Start of expansion, h	75.2 (69.8–90.4)	75.4 (65.4–86.6)	69.0 (66.0–75.9)
Number of blastocysts, n (%)	10 (62.5%)	10 (62.5%)	13 (81.3%)
High-quality embryo, n (%)	8 (50.0%)	5 (31.3%)	10 (62.5%)

*IQR, interquartile range*.

a *Number of embryos from start of culture*.

b *p = 0.065 in comparison with control, Mann-Whitney U-test*.

### Proteomics and secretomics

**Figure 1:**
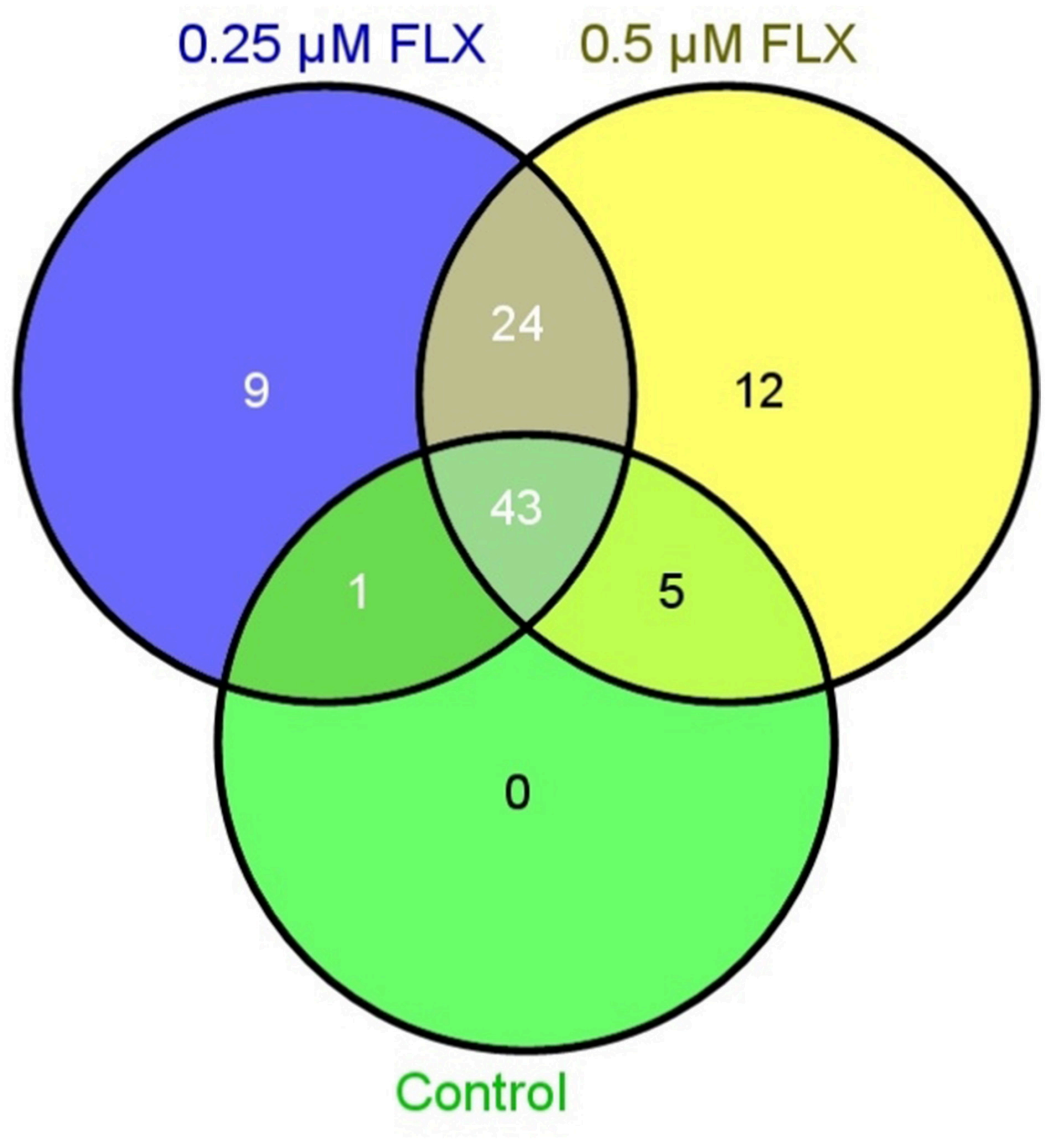
**Venn diagram illustrating the number of proteins detected by mass spectrometry in embryos treated with 0.25 μM fluoxetine (FLX), 0.5 μM fluoxetine and controls**.

A proteome analysis on the human embryos was performed to investigate unique protein expression patterns induced by fluoxetine. Total protein lysate from single embryos, cultured in ordinary medium supplemented with 0.25 or 0.5 μM fluoxetine, were analyzed by use of shotgun mass spectrometry and compared to embryos cultured in ordinary medium. As presented in Table 2 and Figure 1, a number of differentiated proteins were detected in the embryos. In total, 45 proteins were uniquely expressed in fluoxetine-treated embryos, among which 24 were detected in both fluoxetine-dose groups (Table 2). Nine proteins were uniquely expressed by embryos treated with 0.25 μM fluoxetine and 12 unique proteins were detected in embryos treated with the higher fluoxetine dose (Table 2). Only proteins that were detected in at least three out of five embryos in one group are presented. In order to determine the biological relevance of these proteins, an IPA analysis was performed, focusing on proteins that were expressed in embryos treated with 0.5 μM fluoxetine (in total 36 proteins). Three protein networks of relevance were detected in 0.5 μM fluoxetine-treated embryos (Table 3 and Figure S1): (1) Cell Death and Survival, Cellular Growth and Proliferation, Hematological Disease with an IPA score of 23; (2) Cancer, Organismal Injury and Abnormalities, Reproductive System Disease with an IPA score of 18; and (3) Inflammatory Response, Cell Death and Survival, Digestive System Development and Function with an IPA score of 14.

**Table 2:** **Proteins detected by mass spectrometry that were found uniquely in 0.25 μM fluoxetine- and 0.5 μM fluoxetine-treated embryos**.

**0.25 μM fluoxetine**			**0.5 μM fluoxetine**			**0.25 and 0.5 μM fluoxetine**		
**UniProtKB**	**Protein**	**Description**	**UniProtKB**	**Protein**	**Description**	**UniProtKB**	**Protein**	**Description**
P27105	STOM	Erythrocyte band 7 integral membrane protein	P05387	RPLP2	60S acidic ribosomal protein P2	P02790	HPX	Hemopexin
P04217	A1BG	Alpha-1B-glycoprotein	P62917	RPL8	60S ribosomal protein L8	P06748	NPM1	Nucleophosmin
P49411	TUFM	Elongation factor Tu, mitochondrial	P68871	HBB	Hemoglobin subunit beta	P07737	PFN1	Profilin-1
P07954	FH	Fumarate hydratase, mitochondrial	P19338	NCL	Nucleolin	P00738	HP	Haptoglobin
P02749	APOH	Beta-2-glycoprotein 1	P46459	NSF	Vesicle-fusing ATPase	P62937	PPIA	Peptidyl-prolyl cis-trans isomerase A
P43652	AFM	Afamin	P62249	RPS16	40S ribosomal protein S16	Q07021	C1QBP	Complement component 1 Q subcomponent-binding protein, mitochondrial
P02766	TTR	Transthyretin	P04350	TUBB4A	Tubulin beta-4A chain	P04075	ALDOA	Fructose-bisphosphate aldolase A
Q12931	TRAP1	Heat shock protein 75 kDa, mitochondrial	P00441	SOD1	Superoxide dismutase [Cu-Zn]	P00558	PGK1	Phosphoglycerate kinase 1
P04179	SOD2	Superoxide dismutase [Mn], mitochondrial	Q5T2N8	ATAD3C	ATPase family AAA domain-containing protein 3C	Q08257	CRYZ	Quinone oxidoreductase
			P05023	ATP1A1	Sodium/potassium-transporting ATPase subunit alpha-1	Q3ZCM7	TUBB8	Tubulin beta-8 chain
			Q00610	CLTC	Clathrin heavy chain 1	P10696	ALPPL2	Alkaline phosphatase, placental-like
			P67809	YBX1	Nuclease-sensitive element-binding protein 1	P06733	ENO1	Alpha-enolase
						P07910	HNRNPC	Heterogeneous nuclear ribonucleoproteins C1/C2
						Q06830	PRDX1	Peroxiredoxin-1
						P07237	P4HB	Protein disulfide-isomerase
						Q9BRA2	TXNDC17	Thioredoxin domain-containing protein 17
						P60174	TPI1	Triosephosphate isomerase
						P22626	HNRNPA2B1	Heterogeneous nuclear ribonucleoproteins A2/B1
						P62805	HIST1H4A	Histone H4
						P05787	KRT8	Keratin, type II cytoskeletal 8
						P30086	PEBP1	Phosphatidylethanolamine-binding protein 1
						Q99497	PARK7	Protein DJ-1
						P15374	UCHL3	Ubiquitin carboxyl-terminal hydrolase isozyme L3
						P16949	STMN1	Stathmin

*Only proteins that were detected in at least three out of five embryos in one group are presented*.

**Table 3:** **Networks identified by Ingenuity Pathway Analysis (IPA), focusing on the proteins detected in 0.5 μM fluoxetine-treated embryos**.

**IPA networks top 3**	**Proteins**	**IPA score**
Cell Death and Survival, Cellular Growth and Proliferation, Hematological Disease	ENO1, HBB, HNRNPA2B1, HNRNPC, NCL, NPM1, PEBP1, PFN1, PPIA,RPL8, RPS16, YBX1	23
Cancer, Organismal Injury and Abnormalities, Reproductive System Disease	ALDOA, CLTC, P4HB, PARK7, PGK1, PRDX1, RPLP2, STMN1, TPI1, TUBB4A	18
Inflammatory Response, Cell Death and Survival, Digestive System Development and Function	ATP1A1, C1QBP, HP, HPX, KRT8, NSF, PPIA, SOD1	14

Finally, protein secretion from fluoxetine-treated embryos was compared with control embryos. Culture medium in which the different embryos had been cultured was analyzed by use of the Olink Proseek Multiplex Inflammation I^96×96^ Immunoassay. After normalization against culture medium without fluoxetine supplement, a number of proteins were detected above the limit of detection (LOD) (Table 4 and Table S1). Urokinase-type plasminogen activator (uPA) levels were significantly higher in 0.5 μM-treated embryos than in 0.25 μM-treated embryos. For the other proteins, there were either no significant differences between the groups or the number of embryos where the protein could be detected was too limited for statistical analyses (Table 4 and Table S1).

**Table 4:** **Top 10 proteins detected by Multiplex Immunoassay analysis in medium from 0.25 μM fluoxetine-, 0.5 μM fluoxetine-treated and control embryos**.

**Protein**	**UniProtKB**	**Control *n* = 10**		**0.25 μM fluoxetine *n* = 10**		**0.5 μM fluoxetine *n* = 10**	
		***n***	**Median (range)**	***n***	**Median (range)**	***n***	**Median (range)**
uPA	P00749	9	1.30 (0.40–3.20)	10	1.15 (0.30–3.00)	10	2.15 (0.90–3.40)^*^
IL-6	P05231	8	1.35 (0.80– 3.20)	10	1.50 (0.40–2.40)	10	2.05 (0.50–4.90)
ADA	P00813	7	1.70 (1.10–2.30)	5	1.40 (1.20–1.80)	5	1.50 (1.10–1.80)
STAMPB	O95630	7	0.50 (0.40–1.10)	4	0.45 (0.40–0.70)	6	0.55 (0.40–1.00)
CST5	P28325	6	0.10 (0.10–0.20)	3	0.10 (0.10–0.10)	1	0.10 (N/A)
FGF-23	Q9GZV9	5	0.40 (0.30–0.40)	6	0.35 (0.30–0.50)	1	0.30 (N/A)
Beta-NGF	P01138	5	0.60 (0.40–1.50)			2	0.25 (0.20–0.30)
IL-8	P10145	4	0.15 (0.10–5.20)	1	0.10 (N/A)	5	1.30 (0.20–3.20)
IL-10	P22301	4	0.40 (0.30–0.40)	1	0.40 (N/A)	1	0.10 (N/A)
VEGF-A	P15692	3	0.60 (0.10–1.80)	6	0.80 (0.20–2.00)	8	0.85 (0.20–1.70)

* *P < 0.05 compared to 0.25 μM fluoxetine, Mann-Whitney U-test*.

### Immunohistochemical staining of cultured embryos

**Figure 2:**
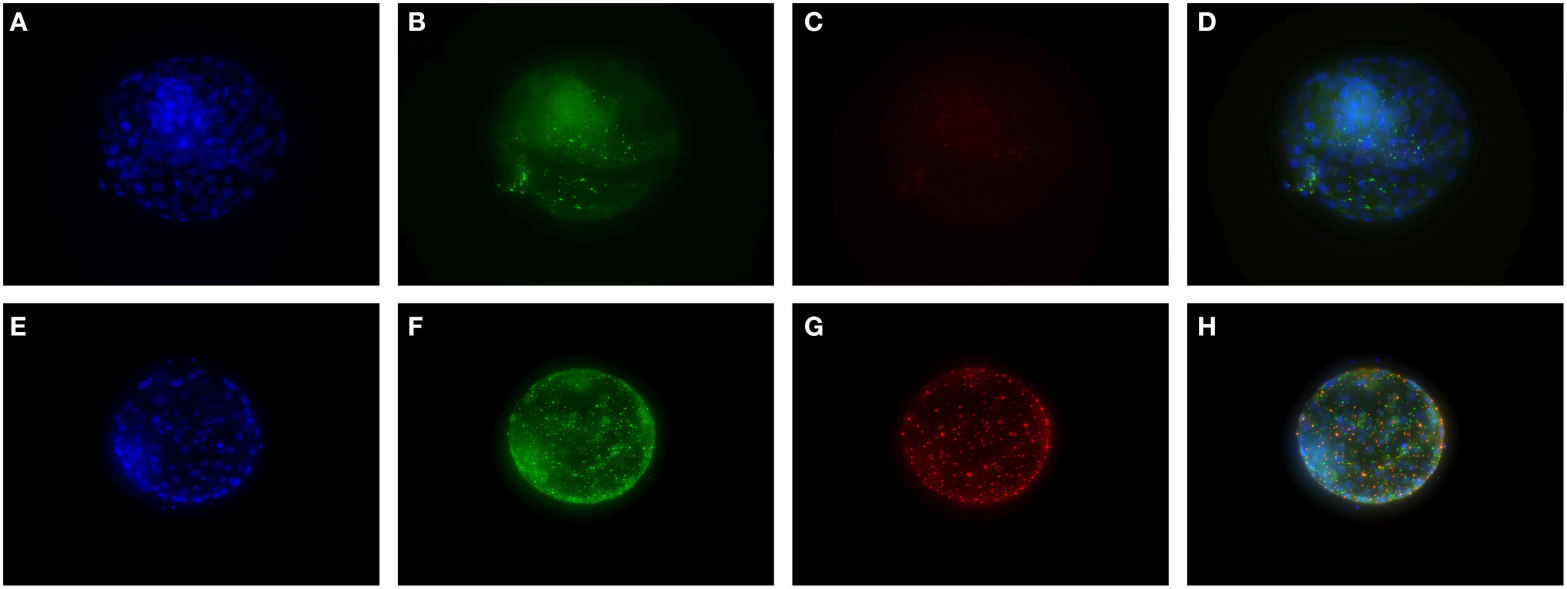
**Immunohistochemical staining of cultured human embryos. (A–D)** are embryos cultured in control medium, **(E–H)** are embryos cultured in 0.5 μM FLX. DAPI staining (blue) shows cell nuclei. Staining for NGF is shown in green and staining for uPA in red. **(D,H)** are overlay pictures.

To validate true presence in human embryos for some of the proteins of interest, immunohistochemistry was performed. uPA and nerve growth factor (NGF) were chosen because of their high ranking in the Multiplex Immunoassay analysis. Furthermore, we have previously found differences in NGF signaling in SSRI-exposed placenta (Kaihola et al., 2015). Staining of uPA and NGF was found in the cytosol and cellular membrane of the trophectoderm of the embryos. NGF was also found in the inner cell mass, whereas uPA had a weak staining in the inner cell mass (Figure 2)

## Discussion

We have previously shown that SSRI treatment during pregnancy affects gene expression and protein levels in the placenta, and pathways of interest for placental function (Kaihola et al., 2015; Olivier et al., 2015b). Based on the established role for 5-HT in embryogenesis (Lauder and Krebs, 1978; Lauder et al., 1981; reviewed in Buznikov et al., 2001), and the fact that serotonin transporters are expressed in early mouse embryos (Amireault and Dube, 2005), we hypothesized that the fetus may also be affected though direct exposure to SSRIs. In this study we demonstrate that fluoxetine has a marginal influence on early human embryo development in culture. Whereas the lower fluoxetine dose had no influence on embryo development compared to controls, embryos treated with 0.5 μM fluoxetine tended to need a shorter time between thawing and start of cavitation. Furthermore, a small dose-response pilot study was conducted before the trial started, where even higher concentrations of fluoxetine (1.0 μM) increased the number of dead embryos. These findings are in line with prior research in mouse embryos (Kim et al., 2012), where it was shown that short-term exposure of 2-cell mouse embryos to fluoxetine increased the percentage of blastocysts via activation of Ca^2+^/calmodulin-dependent protein kinase II (CaMKII)-dependent signal transduction pathways. Furthermore, fluoxetine enhanced mouse embryonic development into blastocysts up to a certain dose, followed by an inhibition of blastocyst formation at higher doses (Kim et al., 2012). In the study by Kim et al. (2012), short-term exposure (6 h) of 2-cell mouse embryos to doses up to 5–10 μM fluoxetine increased the number of blastocysts. However, longer exposure to 5 μM fluoxetine (up to 72 h) resulted in a reduction of embryos that developed into blastocysts. Notably, the doses used in the present study corresponded to one tenth of the doses used in mice, but were chosen to correspond with human physiological umbilical cord and amniotic fluid concentrations of fluoxetine-exposed fetuses (Hendrick et al., 2003; Rampono et al., 2004; Loughhead et al., 2006). From research within the field of IVF, it is known that timing to different developmental stages correlate with embryo quality and with implantation rate after assisted reproduction (Wong et al., 2010; Meseguer et al., 2011; Machtinger and Racowsky, 2013; Kaser and Racowsky, 2014). Although the optimal embryo morphokinetics remains to be settled, i.e., whether accelerated development is good or bad for the implantation rate, the lower fluoxetine dose had less impact than the higher dose on embryo development in our experiments, and this was also true for the embryo protein expression and secretion. For this reason, clinicians should adhere to recent National Institute for Health and Care Excellence (NICE) guidelines (National Institute for Health and Care Excellence, 2014) on SSRI treatment in pregnancy, and prescribe the lowest possible dose in women who need to continue antidepressant therapy when pregnant. However, while the lower dose had less impact, according to what could be detected with the present methods, this finding does not indicate that it is harmless.

The exact mechanism by which SSRI influence early embryo development is not known, although alteration in serotonin levels (Lauder et al., 1981) or activation of CaMKII-dependent signal transduction pathways would be plausible (Kim et al., 2012). Based on our results from the mass spectrometry and proteomics analysis, we hypothesize that pathways of relevance for regulation of cellular growth, proliferation and survival are affected by fluoxetine. The Multiplex Immunoassay analysis revealed significantly increased levels of uPA in culture medium from 0.5 μM fluoxetine-treated embryos. uPA has been shown to be involved in cell proliferation, cell migration (reviewed in Noh et al. (2013) and cellular differentiation. Lino et al. (2014) showed that uPA in complex with its receptor, urokinase-type plasminogen activator receptor (uPAR), is involved in cell signaling during neuronal migration and neuritogenesis in explants from chick embryos. In addition, fluoxetine could be acting through other, hitherto unknown, mechanism(s).

The inconsistency between the Multiplex Immunoassay and mass spectrometry as regards uPA may be due to two different factors. First, the multiplex immunoassay was performed in the culture medium and the mass spectrometry in the embryo, whereby the former method would detect proteins secreted by the embryo and the latter proteins within the embryo. Thus, high levels of uPA in the secretome do not necessarily correspond to the levels detected in the embryo. Potentially, if secretion is high, the embryonic pool of a specific protein could be depleted. Secondly, the Multiplex Immunoassay analysis includes ready-made panels with antibodies directed against target of interest and even though proteins detected by this method were not detected by mass spectrometry, they may very well be expressed in the embryo although not at levels detected by mass spectrometry. However, the detection of uPA by Multiplex Immunoassay analysis was verified by immunohistochemistry of the embryos, where uPA was found mainly in the trophectoderm. The staining for uPA in the trophectoderm goes well with the findings of Khamsi et al. (1996), where uPA was detected in human preimplantation embryos. Both Hofmann et al. (1994) and Teesalu et al. (1996) showed that uPA is expressed in the trophoblast at the maternal-fetal interface in human and mouse implantation sites. Later on in development the trophectoderm will become the placenta, therefore any alterations in placental protein levels may ultimately affect the function of the placenta and then, consequently, also the development of the fetus. Indeed, uPA seems to play a role in trophoblast invasion and in the pathophysiology of preeclampsia (Strickland and Richards, 1992; Zhang et al., 1994; Uszynski and Uszynski, 2011), and may contribute to the increased risk of preeclampsia previously noted in SSRI users (Toh et al., 2009; De Vera and Berard, 2012; Palmsten et al., 2012, 2013). Also, NGF was detected in the embryo trophectoderm which is in line with our previous studies (Kaihola et al., 2015), where the placental NGF levels were increased in placenta from SSRI-treated women.

Our aim with this study was to investigate the effects of pharmacologically relevant levels of fluoxetine on human early embryonic development. For obvious reasons, the number of human embryos that can be used for research is not unlimited. The relatively low number of embryos in this study could have an impact on the results, and this is particularly true for the blastocyst formation and the number of high-quality embryos in each treatment group. However, we noted more than 40 proteins uniquely expressed in fluoxetine-exposed embryo, and also changes in protein expression in the embryo culture medium. Obviously, with a greater sample size, more fluoxetine-induced differences could have been uncovered. The analysis method used for the proteomics has been used in several previous studies within reproductive medicine (Nilsson et al., 2004; Naessen et al., 2010; Hambiliki et al., 2013), but the complexity of the method should be taken into consideration when interpreting and analyzing the results. The embryos used were at a very early stage of development and would in the normal course of events not have implanted in the uterus yet. This means that the placenta has not yet been developed and there is no amniotic fluid or umbilical cord through which fluoxetine could reach and affect the embryo. However, both the oocyte and the embryo could be exposed to fluoxetine, both by the follicular fluid in the ovaries or during the pre-implantation period by secretions in the tuba. Indeed, previous research has indicated that serotonin is found in the follicular fluid and fallopian tubes, at least in female rats (Amenta et al., 1992; Bodis et al., 1993).

Another limitation is that our study cannot elucidate whether the proteins expressed is due to fluoxetine exposure, or the slightly enhanced embryo development, or both. At present, the embryo development proteomics is not known to its full extent, and for that reason, we cannot single out the proteins that would be normally expressed and the ones that are due to toxic effects.

In conclusion, we have studied the effects of fluoxetine on human early embryonic development. We found that fluoxetine has marginal effects on the timing of developmental stages in embryos, but induces expression and secretion of several proteins in a manner that depends on dose. For these reasons, and in line with current guidelines, the lowest possible dose of SSRI should be used in pregnant women who need to continue treatment.

## Author contributions

Conceived and designed the experiments: HK, JO, HÅ, IS. Performed the experiments: HK, FG, JH, KH. Analyzed the data: HK, KH, HÅ, IS. Contributed with reagents/materials/analysis tools: JB, HÅ, IS. Drafting and/or revising the paper, including final approval to publish: HK, FG, JH, KH, JB, JO, HÅ, IS.

## Funding

This research was supported by the Swedish Research Council (grant VR 621-2011-4423) and the Family Planning Foundation, Uppsala.

### Conflict of interest statement

The authors declare that the research was conducted in the absence of any commercial or financial relationships that could be construed as a potential conflict of interest.
