# Supplementary material for: Effects of Fluoxetine on Human Embryo Development
Source: Front Cell Neurosci. 2016 Jun 16;10:160. doi: 10.3389/fncel.2016.00160 (PMC4909759; doi:10.3389/fncel.2016.00160)
Supplement: Supplementary file 1 [file Table1.DOCX]

Supplementary Table

Effects of Fluoxetine on Human Embryo Development

Helena Kaihola*, Fatma Gülen Yaldir, Julius Hreinsson, Katarina Hörnaeus, Jonas Bergquist, Jocelien Olivier, Helena Åkerud, Inger Sundström-Poromaa

*** Correspondence:** Helena Kaihola: helena.kaihola@kbh.uu.se

**Table S1.** Proteins detected by Multiplex Immunoassay analysis and above levels of detection in medium from 0.25 µM and 0.5 µM fluoxetine-treated and control embryos. Proteins detected in at least 3 embryos in total are included.

| **Protein** | **UniProtKB** | **Control**  **n = 10** | | **0.25 µM fluoxetine**  **n = 10** | | **0.5 µM fluoxetine**  **n = 10** | |
| --- | --- | --- | --- | --- | --- | --- | --- |
|  |  | **n** | **Median (range)** | **n** | **Median (range)** | **n** | **Median (range)** |
| uPA | P00749 | 9 | 1.30 (0.40 – 3.20) | 10 | 1.15 (0.30 – 3.00) | 10 | 2.15 (0.90 – 3.40)* |
| IL-6 | P05231 | 8 | 1.35 (0.80 – 3.20) | 10 | 1.50 (0.40 – 2.40) | 10 | 2.05 (0.50 – 4.90) |
| ADA | P00813 | 7 | 1.70 (1.10 – 2.30) | 5 | 1.40 (1.20 – 1.80) | 5 | 1.50 (1.10 – 1.80) |
| STAMPB | O95630 | 7 | 0.50 (0.40 – 1.10) | 4 | 0.45 (0.40 – 0.70) | 6 | 0.55 (0.40 – 1.00) |
| CST5 | P28325 | 6 | 0.10 (0.10 – 0.20) | 3 | 0.10 (0.10 – 0.10) | 1 | 0.10 (N/A) |
| FGF-23 | Q9GZV9 | 5 | 0.40 (0.30 – 0.40) | 6 | 0.35 (0.30 – 0.50) | 1 | 0.30 (N/A) |
| Beta-NGF | P01138 | 5 | 0.60 (0.40 – 1.50) |  |  | 2 | 0.25 (0.20 – 0.30)^#1^ |
| IL-8 | P10145 | 4 | 0.15 (0.10 – 5.20) | 1 | 0.10 (N/A) | 5 | 1.30 (0.20 – 3.20) |
| IL-10 | P22301 | 4 | 0.40 (0.30 – 0.40) | 1 | 0.40 (N/A) | 1 | 0.10 (N/A) |
| VEGF-A | P15692 | 3 | 0.60 (0.10 – 1.80) | 6 | 0.80 (0.20 – 2.00) | 8 | 0.85 (0.20 – 1.70) |
| HGF | P14210 | 3 | 0.50 (0.40 – 0.60) | 2 | 0.40 (0.40 – 0.40) | 4 | 0.50 (0.40 – 0.90) |
| CD5 | P06127 | 3 | 0.20 (0.10 – 0.30) | 2 | 0.15 (0.10 – 0.20) | 3 | 0.20 (0.10 – 0.30) |
| CD6 | Q8WWJ7 | 3 | 0.10 (0.10 – 0.30) | 2 | 0.10 (0.10 – 0.10) | 3 | 0.20 (0.10 – 0.20) |
| MCP-3 | P80098 | 3 | 0.50 (0.40 – 0.50) | 2 | 0.40 (0.30 – 0.50) | 1 | 0.50 (N/A) |
| CCL19 | Q99731 | 3 | 0.30 (0.20 – 0.30) | 1 | 0.30 (N/A) | 3 | 0.30 (0.20 – 0.30) |
| TNFSF14 | O43557 | 3 | 0.10 (0.10 – 0.40) | 1 | 0.10 (N/A) | 3 | 0.10 (0.10 – 0.20) |
| CCL25 | O15444 | 3 | 0.20 (0.20 - 0.20) |  |  | 3 | 0.20 (0.20 – 0.20) |
| CXCL1 | P09341 | 2 | 0.20 (0.10 – 0.30) | 5 | 0.20 (0.10 – 0.50) | 3 | 0.20 (0.10 – 0.30) |
| MCP-4 | Q99616 | 2 | 0.10 (0.10 – 0.10) | 3 | 0.10 (0.10 – 0.10) | 2 | 0.10 (0.10 – 0.10) |
| BDNF | P23560 | 2 | 0.20 (0.10 – 0.30) | 3 | 0.10 (0.10 – 0.10) | 1 | 0.10 (N/A) |
| CSF-1 | P09603 | 2 | 0.15 (0.10 – 0.20) | 3 | 0.10 (0.10 – 0.20) | 1 | 0.20 (N/A) |
| IL-18 | Q14116 | 2 | 0.10 (0.10 – 0.10) | 2 | 0.20 (0.10 – 0.30) |  |  |
| LAP TGF-beta-1 | P01137 | 2 | 0.15 (0.10 – 0.20) | 1 | 0.30 (N/A) | 2 | 0.25 (0.20 – 0.30) |
| MMP-1 | P03956 | 2 | 0.10 (0.10 – 0.10) | 1 | 0.10 (N/A) | 2 | 0.20 (0.10 – 0.30) |
| CCL20 | P78556 | 2 | 0.30 (0.30 – 0.30) | 1 | 0.40 (N/A) | 1 | 0.50 (N/A) |
| IL-1 alpha | P01583 | 2 | 2.80 (1.10 – 4.50) |  |  | 3 | 1.30 (1.20 – 1.80) |
| FGF-21 | Q9NSA1 | 2 | 0.40 (0.40 – 0.40) |  |  | 2 | 0.30 (0.30 – 0.30)^#2^ |
| IL-12B | P29460 | 2 | 0.20 (0.20 – 0.20) |  |  | 2 | 0.25 (0.20 – 0.30) |
| OPG | O00300 | 2 | 0.15 (0.10 – 0.20) |  |  | 2 | 0.20 (0.10 – 0.30) |
| IL-18R1 | Q13478 | 2 | 0.10 (0.10 – 0.10) |  |  | 2 | 0.20 (0.10 – 0.30) |
| IL-10RB | Q08334 | 1 | 0.20 (N/A) | 5 | 0.20 (0.20 – 0.40) |  |  |
| EN-RAGE | P80511 | 1 | 0.40 (N/A) | 4 | 0.30 (0.20 – 0.30) | 1 | 0.30 (N/A) |
| IL-15RA | Q13261 | 1 | 0.40 (N/A) | 3 | 0.40 (0.40 – 0.50) |  |  |

* p<0.05 compared to 0.25µM FLX, Mann-Whitney *U* test

#1 p=0.053, #2 p=0.083 compared with control, Mann-Whitney *U* test
