# Supplementary material for: Effects of Fluoxetine on Human Embryo Development
Source: Front Cell Neurosci. 2016 Jun 16;10:160. doi: 10.3389/fncel.2016.00160 (PMC4909759; doi:10.3389/fncel.2016.00160)
Supplement: Supplementary file 2 [file Image1.PDF]

Supplementary Figure

Effects of Fluoxetine on Human Embryo Development

Helena Kaihola*, Fatma Gülen Yaldir, Julius Hreinsson, Katarina Hörnaeus, Jonas Bergquist, Jocelien Olivier, Helena Åkerud, Inger Sundström-Poromaa

*** Correspondence:** Helena Kaihola: helena.kaihola@kbh.uu.se

Figure S1. Networks identified by Ingenuity Pathway Analysis (IPA) focusing on the proteins detected in 0.5 µM fluoxetine-treated embryos. Proteins marked in red are proteins detected in the embryos using mass spectrometry. Unmarked proteins are proteins in the networks with which the identified proteins interact. **(A)** IPA network 1, **(B)** IPA network 2, and **(C)** IPA network 3.

A)

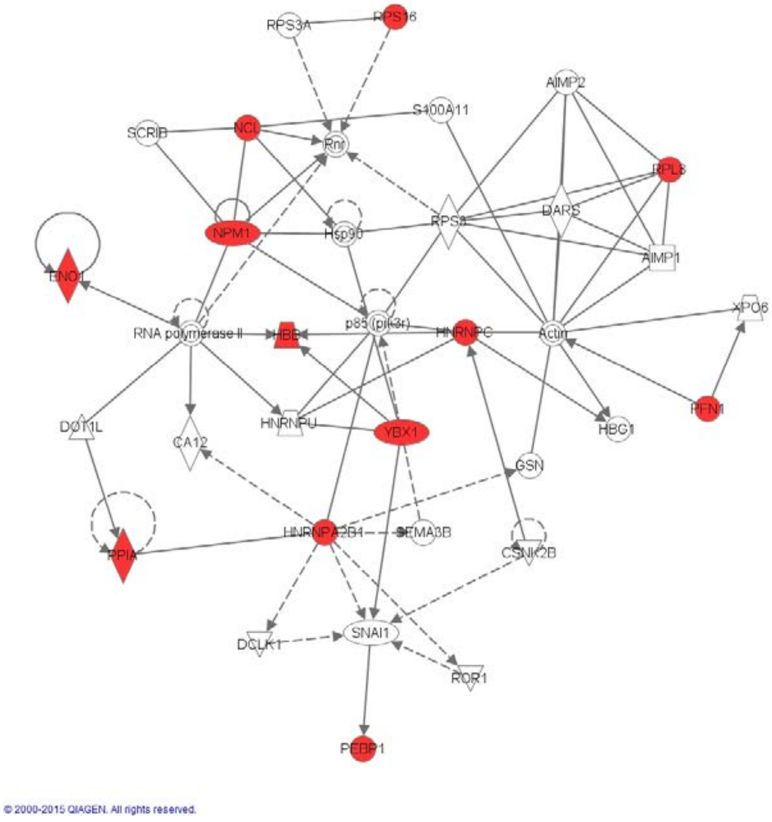

B)

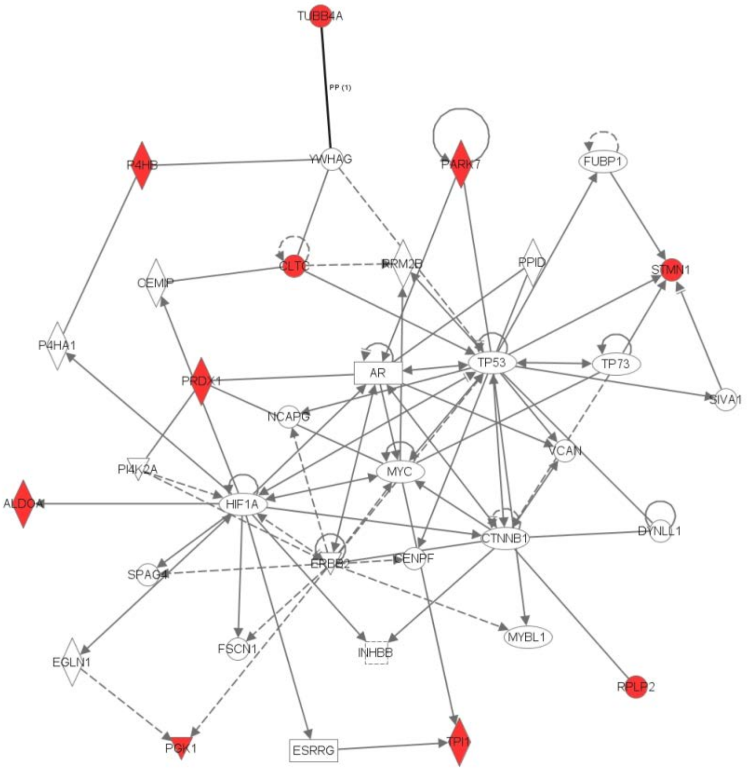

© 2000-2015 QIAGEN. All rights reserved.

C)

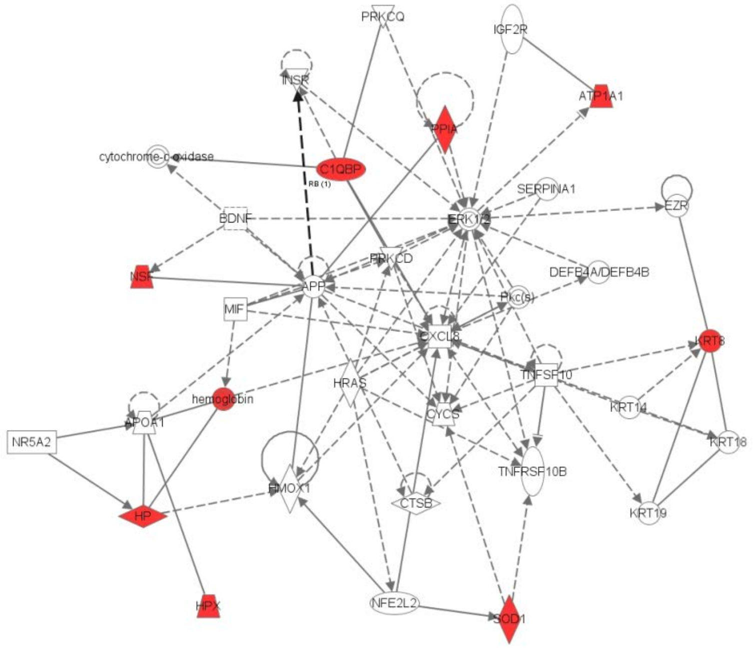

© 2000-2015 QIAGEN. All rights reserved.
